# The Dimeric Proto-Ribosome: Structural Details and Possible Implications on the Origin of Life

**DOI:** 10.3390/ijms10072921

**Published:** 2009-06-30

**Authors:** Ilana Agmon

**Affiliations:** The Department of Structural Biology, Weizmann Institute, Rehovot 76100, Israel; E-Mail: agmon1@gmail.com; Tel. 972-4-8372956; Fax: 972-77-664-7774

**Keywords:** proto-ribosome, origin of life, ribosome symmetrical region

## Abstract

A symmetric pocket-like entity, composed of two L-shaped RNA units, encircles the peptide synthesis site within the contemporary ribosome. This entity was suggested to be the vestige of a dimeric proto-ribosome, which could have formed spontaneously in the prebiotic world, catalyzing non-coded peptide bond formation and elongation. This structural element, beyond offering the initial step in the evolution of translation, is hypothesized here to be linked to the origin of life. By catalyzing the production of random peptide chains, the proto-ribosome could have enabled the formation of primary enzymes, launching a process of co-evolution of the translation apparatus and the proteins, thus presenting an alternative to the RNA world hypothesis.

## 1. Introduction

Evolution of translation is a key question in understanding the emergence of life. In translation, the complicated process by which genetic information is translated into proteins, amino acids are polymerized into polypeptide chains that in turn fold into proteins. This takes place at the ribosome, a large, complex, universal ribonucleoprotein catalytic machine, composed of a large and a small subunit (50S and 30S in prokaryotes). The ribosome central task, peptide bond formation, occurs in the active site of the large subunit, the Peptidyl Transferase Center (PTC), which, at least in prokaryotes, is composed solely of RNA. Being a ribozyme can increase the likelihood that the ribosome remains active under protein-related stress conditions, enabling it to reproduce defective proteins.

**Figure 1.**
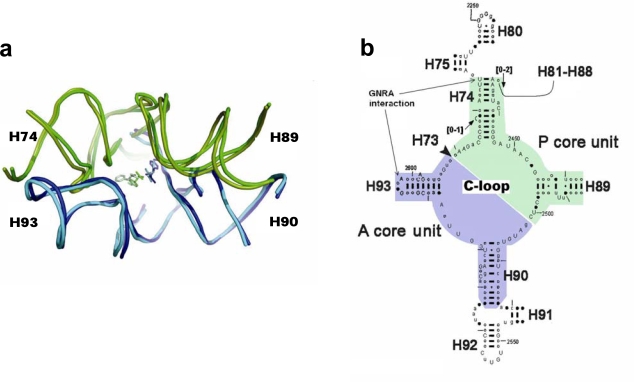
The proto-ribosome: (a) The suggested remnant of the proto-ribosome as appears within the ribosomal large subunit, projected approximately along the symmetry axis. The pocket was obtained by dimerization of the A-, P- core units (marked in blue and green, respectively. Same color scheme maintained in all figures), shown by their RNA backbone, with models of the amino acid reactants, as found in the crystal structure of the ribosomal large subunit of bacteria (*Deinoccocus radiodurans*, D50S, PDB code 1NJP) and archaea (*Haloarcula marismortui*, H50S, PDB code 1VQN) complexed with substrates mimicking the tip of tRNA 3′ end. The P-site amino acid in the D50S structure was derived from the A-site amino acid by applying the rotatory motion [5,6]. (b) 2D diagram of the symmetrical region from *E. coli* drawn in a manner portraying the 3D symmetry. The scheme shows phylogenetic conservation in 930 species from three domains and two organelles [12]. Nucleotides marked by capital A, C, G, and U are more than 98% conserved, while those depicted as points are less than 90% conserved. The 2D scheme of the proto-ribosome remnant, constructed from the two symmetry related ribosomal core units, each composed of two stems connected via a single stranded region, is shown on colored background.

The emergence of the elaborate contemporary ribosome by mere chance is highly unlikely. It is conceivable that the modern ribosome evolved from a far simpler proto-ribosome [1], which could catalyze only non-coded peptide bond formation between two small substrates and allowed simple elongation. In the contemporary ribosome, peptide bond formation takes place within the PTC, in a zone devoid of interactions with elements involved solely with coded peptide synthesis, *i.e.* with the tRNA’s stems and elbow and with the various elongation factors. This zone, which forms a pocket-like RNA element (Figure 1a), was suggested [2,3] to be the vestige of a primordial template capable of accommodating single amino acids or short peptidyl chains, catalyzing peptide bond formation and achieving elongation rates exceeding those of non-catalyzed reactions, *i.e.* of the proto-ribosome.

Based on the pocket-like structure found within the ribosome, the proto-ribosome was suggested to be a dimer composed of two L-shaped RNA units [3], which, being part of the ribosomal symmetrical region [4,5] (Figure 1b), are similar to each other in fold and in nucleotide conformation but differ in sequence. Symmetrical association of the two units, named the A- and P- ribosomal core units, enables a favorable mutual accommodation of the reactants [3], equivalent to the manner by which the reactants are positioned on the ribosome [5–8], in a stereochemistry favorable for peptide bond formation. The dimeric proto-ribosome was suggested to have been the starting point for the evolution of the modern complex translational machine- the ribosome [2,3].

An evolved dimeric proto-ribosome, capable of stably accommodating larger substrates, would have involved the addition of the A-, P- sites (H92, H80, respectively, which accommodate the A-, P- substrates in the contemporary PTC). The dimer of the two core units, together with the A-, P- sites, emerging from far ends of H74 and H90 for P-, A- core units, respectively [3], assemble the entire symmetrical region (Figure 1b). An entity similar to this evolved dimeric proto-ribosome, which contains the complete symmetrical region attached to the non-symmetrical part of H75, was recently suggested to be the proto-ribosome, based on the role of the A-minor interactions in the evolution of the ribosome [9]. Larger entities of about 200 nucleotides, including the vicinity of the contemporary large subunit active site, were previously suggested to be associated with the proto-ribosome [10,11].

Here the structure of the dimeric proto-ribosome is discussed in details and a possible relevance to the origin of life is suggested.

## 2. The Structure of the Dimeric Proto-Ribosome

The dimeric proto-ribosome was a pocket assembled from two L-shaped RNA core units [3] (Figure 1a). Within the contemporary ribosome, the vestige of each unit contains about 60 nucleotides, which are conserved throughout kingdoms and organelles [5]. The core unit is a stem-elbow-stem structure, composed of two helices (H90 and H93 in the A-core unit, H74 and most of H89 in the P-core unit), joined by an elbow region created via a network of hydrogen bonds involving nucleotides from the Central loop of domain V (C-loop) (Figure 1b). The stems of the original core unit monomers were closed by stem loops, but its vestige within the contemporary ribosome lacks three out of the four expected loops (Figure 1a, b), probably because the ribosome expanded during evolution from tips of the helices. The additional loop nucleotides would have increased the length of a core unit sequence up to 70-mer.

**Figure 2.**
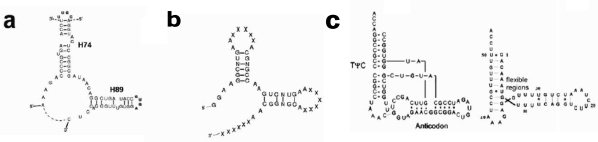
Secondary structure of L-shaped RNA molecules: (a) P-core unit from *E.coli*. Nucleotides marked by red letters were artificially added to the original ribosomal sequence, to complete truncated helices. H89 was closed by a GUGA tetraloop due to the symmetry relation to the GUGA loop of H93. Continuous and broken lines mark the two possible location of the 3′;5′ ends *i.e.* the elbow option and the tRNA-like options, repectively. (b) Right-angle tectoRNA molecule having the 3′:5′ at the elbow region [14] (c). L-shaped scheme of canonical tRNA (left) and mitochondrial tRNA possessing a flexible elbow angle (right) [21], having the 3′:5′ at the acceptor stem end.

**Figure 3.**
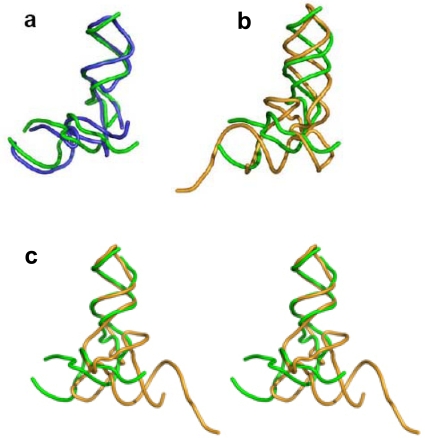
3D structure of L-shaped RNA molecules: H89 is shown in a vertical positioning (a) Overlap of the A- and P- core units from the structure of D50S (PDB code 1NKW), obtained by a rotation of 178.6° around the symmetry axis (LSQKAB program [27]). The projection direction is perpendicular to that shown in Figure 1a. (b) Comparison of the size and shape of the P- core unit (in green) and the tRNA (in light brown). (c) The anticodon helix of the tRNA molecule is overlapped on H89 of the P- core unit (stereo view).

The structure of the ribosomal core unit can be analyzed by comparing it to the known structures of other L-shaped molecules. The secondary structures of the core unit (Figure 2a), the tectoRNA molecule with a right angle motif [13–15] (Figure 2b) and the tRNA (Figure 2c), show that the 3′:5′ ends can be found in the elbow region, as in the tectoRNA, or at the end of one of the two arms, as in the tRNA. The 3′:5′ ends of the A-, P-core units within the ribosome, are located at the elbow region formed by C-loop single stranded segments, equivalently to the tectoRNA structure. The 3′ and the 5′ ends are, however, adjacent in the ribosome three-dimensional structure (Figure 3a), allowing for the possibility that the current 3′:5′ ends could have been covalently bonded in the proto-ribosome. This may indicate the possible prebiotic permutation of the 3′:5′ ends to the tips of H74 and H90 (Figure 2a) for the P-, A-core units, respectively, resulting in a tRNA-like structure.

Secondary structures of short RNA sequences, similar in size to the tRNA structure, can be reliably predicted by free energy minimization [16] and can add support to conclusions drawn from the 3D structure. Using Mfold [17,18] (version 3.2), the thermodynamically favored 2D structures of the A- and the P-core unit sequences, in a 3′:5′ tRNA-like form and in a 3′:5′ elbow form (Figure 2a), were predicted. The calculation was performed on the core unit sequences as found in the structures of D50S, H50S and *E.coli* ribosomes (applicable also to *Thermus thermophilus*, having identical sequence to D50S in the core units region). Although no two of the sequences gave identical 2D structure, the calculated structures were consistently arranged as a stem-elbow-stem fold. The average free energy of the 3′:5′ elbow form was in the range Δ G=26 ± 3 kcal/mole, indicating the self-folding and stability of the obtained structures [3], while the same sequences arranged in the 3′:5′ tRNA-like form were less stable by 1.3 kcal/mole on average. The calculation was carried out under default conditions, *i.e.* temperature of 37 °C and ionic conditions fixed at [Na^+^]=1 M, with no divalent ions. If the prebiotic conditions were similar to the Mfold default ones, the 3′:5′ elbow form may have been the abundant one. Under much different environmental conditions, however, a tRNA-like structure could have been the more stable fold of the core unit.

A 3D comparison of the L-shaped structures reveals that while the core unit elbow angle is stabilized at about 105° (Figure 3a), the elbow angle for the two other L-shaped molecules is about 90°. Canonical tRNAs possess an intricate elbow region which maintains the elbow angle at about 90°, adapted to optimal interaction with the contemporary ribosome. The tRNA elbow region is, however, the part most prone to induced conformational changes: 1) the tRNA elbow was found to go through a complete reorganization to allow the accessibility of modification enzymes [19]. 2) The non-active conformer of tRNA, which is obtained from the canonical one under ionic strength change, preserves the structure of anticodon and the acceptor stems while altering the elbow region structure [20]. 3) Mitochondrial tRNAs possess an obtuse elbow angle [21,22]. 4) The tRNA elbow is the first to dissolve under elevated temperature [23]. In the tectoRNA structures, sequence variations in the linker connecting the two helices, has led to enhanced flexibility of the elbow region, which was correlated with higher affinity towards dimerization [24]. The above examples indicate that the stability of the elbow region in an L-shaped molecule is lower than the rest of the structure and that inter-stem angles deviating from 90° exist in L-shaped molecules, possibly promoting dimerization.

The core unit and the tRNA have comparable size and outline (Figure 3b). In particular, if one tRNA helix is overlapped with one of the core unit helices, their remaining helices point to opposite directions (Figure 3c). The tRNA is widely accepted to be a relic from the prebiotic world [25,26], while the core unit is implicated in prebiotic peptide bond formation [3]. These two forms of L-shaped molecules could have existed, side by side, in the prebiotic environment, being later recruited for their separate roles in translation. The later recruitment of existing prebiotic molecules towards evolving tasks is supported by the variety of roles tRNA-like molecules perform in replication [26].

RNA molecules tend to form stable dimers spontaneously [28–30]. It is therefore likely that two core units could have self-assembled under prebiotic conditions to form a dimer. The presence of divalent metal ions could have been a prerequisite, since they are crucial for the correct folding, stability and dimerization of RNA molecules *in vitro* [20,31–33] and the formation of the proto-ribosome, which preceded life, is equivalent to an in-vitro reaction. In the high resolution structures of the modern ribosome (PDB codes 1FFK, 1NKW, 2AW4), metal ions are found near the single stranded segments of the C-loop, comprising the core unit elbow, but none are involved in the dimer-forming interactions between the two core units. This does not necessarily apply to the proto-ribosome, as the possible role of the metal ions in gluing the two units within the prebiotic dimer could have been substituted by the stabilizing effect exerted on the embedded dimer by the modern ribosome bulk.

**Figure 4.**
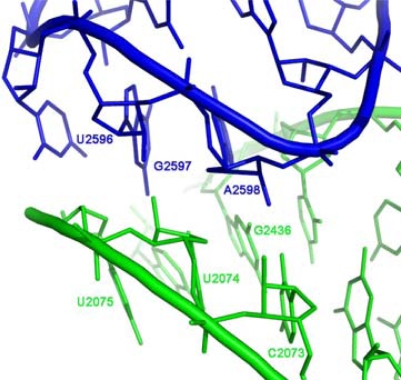
GNRA interaction motif joining the A-, P-core units within the contemporary ribosome (D50S PDB code 1NKW). The GUGA tetra stem loop of H93 (nucleotides G2595-A2598) interacts with a receptor region comprised of nucleotides from H74 (nucleotides G2436:C2073, A2435:U2074, U2075). A2598 makes an A-minor interaction with G2436:C2073.

In the contemporary ribosome, the association of the two core units around the approximate 2-fold rotational symmetry axis is partially achieved by a GNRA interaction motif. The GNRA motif (N=any nucleotide; R=purine) is an abundant non-Watson-Crick RNA-RNA interaction, in which a terminal GNRA loop recognizes a helical receptor region [34], so mediating RNA tertiary interactions [35] and stabilizing symmetrical dimers of RNA molecules [33]. Within the ribosome, the GNRA interaction which joins the A-, P- core units, takes place between the far ends of the stems (Figure 1b), involving the GUGA stem loop of H93 and a receptor region on H74 (Figure 4) and is highly conserved. The tetra-loop nucleotides G2595, R2597 and A2598 are all 100% conserved, the receptor base pair involved in the A-minor interaction, C2073:G2436, is about 95% conserved, A2435:U2074 is 100% conserved and U2075 is 99% conserved [12].

Due to the 2-fold symmetry of the region, a second GNRA interaction motif is expected to exist between the two core units, *i.e.* between a tetra stem loop of H89 and a receptor area on H90. In the modern ribosome, however, this interaction is composed solely of a single hydrogen bond [5] as a result of the lengthening of H89 by a non-symmetrical extension (Figure 1b), probably due to a later evolving task.

An equivalent form of symmetrical self-dimerization, obtained via GNRA tertiary interactions, is used to construct various forms of tectoRNA dimeric particles [33]. This type of dimerization was found to contribute to the stability of the molecules, implying that self-assembly of core units into symmetrical dimers through GNRA interaction is an energetically favorable process that could have occurred spontaneously in a primordial world, generating the dimeric proto-ribosomes.

Considering that only the extant four RNA nucleotides, in equal frequencies, existed when the proto-ribosome emerged, about 3% of the ancient core units would have had the suitable tetra-loop sequence to be involved in a GNRA interaction, and the requirement for the right receptor would have further decreased the probability for such an interaction. Biochemical studies of the interactions between GNRA tetra-loops and RNA helices indicate, however, that both the loop and the receptor can tolerate high degree of sequence variability without eliminating binding affinity or specificity [36,37], suggesting a realistic probability for the formation of the symmetrical dimers through GNRA interactions.

The requirement for dimerization via a GNRA interaction can be reduced to dimerization through an A-minor interaction [38,39], which is part of the GNRA motif (Figure 4). Such a suggestion correlates with the recent hypothesis that new RNA elements were added to prior versions of the ribosome by A-minor interactions [9]. From sequence point of view the sole requirement for dimer formation through an A-minor motif, is an Adenine at the fourth position of the tetra stem loop, as such adenine forms an A-minor interaction with any Watson-Crick base pair, although it prefers a C:G pair [39]. Consequently, if dimerization occurred through an A-minor motif, about 25% of the core unit population at the prebiotic time would have had stem loops appropriate for symmetric dimerization.

Experimental work conducted in Ada Yonath’s group, mainly by Chen Davidovich, indicated that some short RNA sequences resembling those observed in the current ribosome are capable of forming dimers spontaneously. Moreover, the variation in the stems length of the ribosomal core units was found to hamper dimerization, suggesting that the dimers have a ‘pocket-like’ structure [3, Davidovich *et al*., to be published]. These results support the notion that self-folding of oligonucleotides and self-assembly of core units into pocket-like entities are energetically favorable processes that could have occurred spontaneously in a primordial world, producing the dimeric proto-ribosomes.

Consistent with the expectation for a continuous evolutionary path from the proto-ribosome towards the modern ribosome, the dimeric proto-ribosome is assumed to have accommodated the reacting amino acids in the same manner as the modern ribosome does [3], thus allowing the atoms participating in the nucleophilic attack to interact effectively. The reacting amino acids in the ribosome are accommodated against nucleotides from the single stranded C-loop segments, requiring that this part of the structure should be identical in any efficient proto-ribosome, to ensure preservation of the exact peptide synthesis mechanism. The loop-receptor nucleotides involved in the dimer forming interactions should be conserved as well, but differences in the stem’s sequence may be tolerable, as long as the inner surface of the pocket is retained. A G:C base pair in the stems may, for example, be exchanged by A:U without causing functional changes. Indeed, in the contemporary ribosome, the single stranded C-loop nucleotides are nearly all highly conserved, while the stem nucleotides, which constitute the majority of the proto-ribosome structure, show base-pair variation among species (Figure 1b). The limited requirement for sequence specificity in the dimeric proto-ribosome, is best presented by the symmetric A- and P- core units as found within the contemporary ribosome, which, in spite of the significant 2D (Figure 1b) and 3D (Figure 3a) resemblance, are hardly related sequentially. In *E. coli*, for instance, only 38% of the symmetrical mates have identical nucleotides type, relative to 25% expected from random sequences. The minor sequential match could have been affected to some extent by later mutations, but it still points to an inherent limited sequence specificity required from the unit core monomer and thus- from the dimeric proto-ribosome.

## 3. Discussion

Two prerequisites are obligatory for the prebiotic formation of the dimeric proto-ribosome: the primordial existence of amino acids and of oligonucleotides of about 35 mer (if the core unit emerged by association of two hairpins, equivalently to the suggestion for tRNA-like molecule [40–42]), or of about 60–70 mer, if the core unit emerged as a whole. A core unit could have then spontaneously dimerized, in a symmetrical manner, with a second core unit, similar in fold but most likely having a different sequence, as the probability of encountering a sequentially identical core unit would have been infinitesimal. Heterodimerization could have been beneficial for peptide bond formation, as it allows small deviation from perfect symmetry. Perfect symmetry would have posed the amino N of one reactant against the amino N of the second reactant, and not facing the carbonyl C, in a mutual conformation that is somewhat remote from the optimal one for peptide bond formation. Small deviations prom perfect symmetry, such as found within the ribosome, where the amino acid at the P-site is 2A deeper into the tunnel direction relative to the A-site amino acid [5,6], posing the reactants in a favorable stereochemistry for peptide bond formation [6–8], are better associated with a heterodimer.

This simple apparatus was probably the smallest possible dimeric proto-ribosome, as indicated by association of the core units into dimers through the tips of their stems (Figure 1b). The loop-receptor interaction, which forms the symmetrical dimerization of the tectoRNA molecules, was found to be critically dependent upon the mutual helical twist of the interacting stems [24]. In order to preserve the proto-ribosome inner pocket, which accommodates the reactants, the mutual conformation of the stems should be retained. Conservation of the interaction motif is only possible if the length of the stems is shortened by a full helical turn, which, for A-form RNA, holds 11 base pairs. Shortening of the core unit stem length by 11 base pairs will eliminate the stems completely (Figure 2a), implying that the dimeric proto-ribosome was the minimal one feasible for this kind of apparatus.

The dimeric proto-ribosome was implicated in catalyzing peptide bond formation and achieving elongation rates exceeding those of the non-catalyzed reactions, but it may have also been involved in an additional task. In-vitro catalysis of protein folding was identified in domain V of 23S rRNA [43]. The nucleotides found to cross-link to the unfolded peptide chain are in part included in the vestige of the dimeric proto-ribosome within the ribosome and others are vicinal, but exterior to the initial dimer. As the identity of the nucleotides directly involved in the folding catalysis is not known, it is possible that the observed phenomenon is a reminiscent of catalytic folding ability the dimeric proto-ribosome possessed in the prebiotic era, indicating that beyond catalyzing non-coded polypeptide synthesis, the proto-ribosome may have also assisted polypeptide folding.

The hypothesis that the proto-ribosome was a dimeric pocket-like structure whose vestige is still embedded at the active site of the modern ribosome [2,3] offers considerable explanatory power. To begin with, the dimeric proto-ribosome, by its capability to emerge spontaneously and catalyze the formation of random peptide chains, provides a simple and feasible starting point to the complex and extremely important mechanism of modern translation [3]. The dimeric structure of the proto-ribosome can also explain the existence of a symmetrical region within the modern ribosome, which lacks any other symmetry. This structure may be related to the origin of chiral discrimination in proteins. The PTC of the modern ribosome is suggested to prefer l- to d- amino acids due to the enhanced steric hindrance exerted on the d conformation [44,45]. As the dimeric proto-ribosome is proposed to be still embedded in the modern ribosome, it may have favored l- over d- amino acids in the prebiotic era as well, pointing at a possible relevance to origin of chirality in proteins.

Above all- the current hypothesis may offer an alternative way to disentangle the egg and chicken paradox concerned with the origin of life. The paradox, *i.e.* the dependence of proteins on RNA for their formation on the one hand, and the dependence of RNA replication on proteins in the present form of life, led to the suggestion of an RNA world, which preceded life as we know it [46–48], where RNA acted as both the catalytic tool and the heredity source. The dimeric proto-ribosome could have emerged and functioned within an RNA world, where RNA itself, maybe with the help of co-factors, guarantied its production. Alternatively, it could have emerged spontaneously, before any replicative molecular system existed.

The capability of the dimeric proto-ribosome to spontaneously emerge from random oligonucleotides of sufficient length relies on two characteristics: the energetic down-hill processes of formation (folding and dimerization) and the rather limited sequence specificity required from the oligonucleotides involved in the production of the dimeric proto-ribosome.

Consequently, if oligonucleotides of sufficient length existed in the prebiotic era, the proto-ribosomes would form spontaneously, possessing, according to their sequence, diverse peptide synthesis efficiencies. They would have produced a variety of random peptides, whose sequence depended on the distribution of the various amino acids in their vicinity. Such peptide chains would have accumulated in larger amounts in the vicinity of “efficient” RNA dimers, increasing the probability that some of the randomly produced peptidyl chains, which exceeded 30 residues in length, being therefore sufficiently long to allow stable folding [49], will fold into a structure possessing weak catalytic abilities of some kind. Once a protein with some polymerase activity emerged by chance, possibly similar to the conserved palm region of the modern polymerase which contains the catalytic site [50,51], it could have functioned as a primitive RNA-replicating enzyme, copying the original proto-ribosome. The limited accuracy expected of such an initial copying enzyme should not have hindered further evolution, as the dimeric proto-ribosome could have tolerated some sequence variability without impairing its function. Similarly to emergence of the primal replicating enzyme, a folded peptidyl chain with weak synthetase activity could have emerged as well, randomly bonding amino acids and nucleotides together, producing larger potential substrates that could have later attached more stably to the proto-ribosome, launching a process of co-evolution of the translation apparatus and the proteins. The association of primordial machinery for peptide synthesis and an RNA replicase that could have been either a catalytic RNA or an early peptide product, was already suggested to lead to the development of progenotes incorporating both of these features in one enclosure [52].

The primary molecular system suggested here to initiate the transition from the inanimate world towards life, i.e. the dimeric proto-ribosomes and the initial enzymes, might offer some advantages over the RNA world hypothesis:

1. An RNA-protein preliminary system would have naturally evolved into an RNA-protein world without the need to shift from an RNA world into an RNP world, as the RNA world hypothesis entails.
2. The limited specificity of the dimeric proto-ribosome sequence increases the probability of randomly obtaining the suggested proto-ribosome, compared to a fixed-sequence ribozyme of the same size, and allows tolerance to the rather poor RNA copying abilities that the initial replicase (ribozyme or enzyme) would have probably had.
3. The chemical versatility and efficiency of enzymes should have been beneficial in promoting the emergence of life.

Further speculation could maintain that there may have been more than a single proto-ribosome involved in the emergence of life, as some sequence variations could have existed among equally efficient proto-ribosomes, allowing more than one proto-ribosome to be retained by evolution, possibly correlated with the later phylogenetic diversification.

The suggested chemical prebiotic process, originating from an oligonucleotide, proceeding via a self-folded core unit that is more stable then the oligonucleotide, into a self-assembled dimer which is more stable than the single core unit, is an energetically downhill process and could have therefore occurred without any enzymatic aid. The process, however, is suggested to produce proto-ribosome pockets with different levels of protein synthesis efficiency, depending on their different sequences, thus having varying chance of survival. This is a distinctive application of Darwin’s principle of variation and selection, a principle that is usually applied to living organisms. In the scenario suggested here, the sequence variation emerged from the randomness of the RNA chains building the proto-ribosomes rather than from mutations, and was therefore a prebiotic phenomenon. Selection of the fittest proto-ribosome, which led to its survival, would have already been a life process. The appearance of the dimeric proto-ribosome may have therefore been the transition between thermodynamic selection, applicable to chemical reactions, and Darwinian selection, applicable to living organisms, thereby initializing the emergence of life.

## 4. Conclusions

The dimeric proto-ribosome, which was probably the minimal one feasible for this kind of apparatus, could have offered a fitted template for the favorable positioning of small substrates, allowed primitive elongation and maybe assisted polypeptide folding, so catalyzing all the aspects of non-coded prebiotic protein synthesis. Limited variations in the sequences of equally efficient proto-ribosomes may have been possible in principle, potentially correlated with later evolved phylogenetic diversification. By catalyzing random polypeptide chains, suggested to result in the emergence of the primal enzymes, these proto-ribosomes may have initiated the co-evolution of the translation apparatus and the proteins, possibly rendering surplus the need for an RNA world. This apparatus, on the border between matter and living organisms, could have therefore been the starting point of our contemporary forms of life.
